# Beware of Sealing Film of Petri Dishes!—Alters the Expression of a Large Number of Genes

**DOI:** 10.3390/ijms26125484

**Published:** 2025-06-07

**Authors:** Yun Ma, Fang Li, Xuyang Wang, Qingpeng Sun, Ronghuan Wang, Jiuran Zhao

**Affiliations:** 1 Maize Research Institute, Beijing Academy of Agriculture and Forestry Sciences, Beijing 100097, China; mayun@baafs.net.cn (Y.M.); lif0615@163.com (F.L.); 13733761615@163.com (X.W.); zhaojiuran@baafs.net.cn (J.Z.); 2 College of Plant Science and Technology, Beijing University of Agriculture, Beijing 100096, China

**Keywords:** sealingfilms, *Arabidopsis* seedlings, transcriptome, CO_2_ concentrations

## Abstract

*Arabidopsis* seedlings grown in Petri dishes sealed with PE plastic wrap, PP parafilm, or NF surgical tape showed differences in growth, with PE plastic wrap resulting in a smaller size and fresh weight, followed by PP parafilm, compared to unsealed or NF surgical tape-sealed dishes. To investigate the basis of these phenotypic changes, transcriptome sequencing was performed. The results indicated that seedlings in dishes sealed with PE plastic wrap and PP parafilm exhibited over 1000 differentially expressed genes (DEGs) at 7 days. By 14 days, the number of DEGs had increased to over 2000 for each sealed condition. GO analysis showed that DEGs were commonly enriched in biological processes associated with the response to hypoxia under PE plastic wrap and PP parafilm sealing at both 7 and 14 days, as well as under NF surgical tape at 14 days. While O_2_ levels showed no significant differences between sealed and unsealed conditions, CO_2_ concentrations were notably lower in plates sealed with PE plastic wrap and PP parafilm. Furthermore, specific genes related to reduced size and delayed growth under sealed conditions were identified. In summary, sealing films negatively affect seedling growth, leading to significant shifts in gene expression profiles.

## 1. Introduction

**Figure 1:**
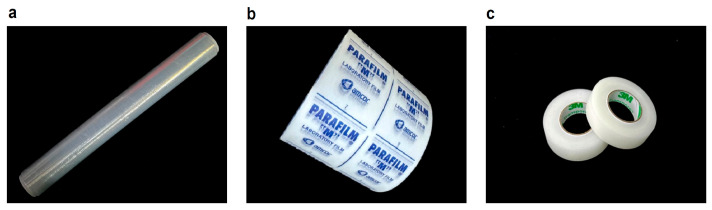
Sealing film commonly used in laboratories: (**a**) Plastic wrap. (**b**) Parafilm. (**c**) Surgical tape.

*Arabidopsis thaliana* is a widely used model plant for genetic and molecular biology research [1]. Since its genome was sequenced in 2000 [2], over 80,000 research papers have utilized *Arabidopsis* as an experimental model (based on PubMed data up to March 2025), with more than 5000 of these studies involving sterilized *Arabidopsis* seeds and seedlings grown on agar-based media (Supplementary Data Set 1). A typical procedure includes surface sterilizing the seeds, sowing them on agar medium, and chilling at 4 °C for 1–3 days. For hypocotyl growth observations, plates are kept in darkness, while for general seedling growth, they are transferred to a growth chamber with a 16 h light/8 h dark cycle for 1–2 weeks. Seedlings may then be transplanted to soil for continued growth or for transgenic experiments or subjected to phenotypic analysis under stress conditions like low temperature or high salinity. Root-related phenotypes are observed by positioning the plates vertically [3,4,5]. An often-overlooked detail in these methods is the sealing of Petri dishes after sowing seeds on solid media. Sealing maintains sterility and humidity inside the plates, a necessary step, especially for vertically positioned plates. Parafilm is commonly used for this purpose, although some labs use plastic wrap or surgical tape (Figure 1). While sealing Petri dishes is routine, few researchers have questioned whether this practice might pose risks to plant growth.

Recently, while preparing to apply stress treatments to two-week-old *Arabidopsis thaliana* seedlings grown on an agar-based medium, we observed an unexpected phenomenon: Seedlings of the same genotype, sterilized for the same duration and sown at identical densities, displayed significant differences in size and developmental stage depending on the type of sealing film used. This variation occurred despite placing the Petri dishes side by side under identical temperature, humidity, and light conditions. To investigate the effects of different sealing materials—parafilm; plastic wrap; and surgical tape—on *Arabidopsis* growth; we conducted transcriptome sequencing (RNA-seq) on seedlings grown under each sealing condition; as well as on unsealed controls. We also measured O_2_ and CO_2_ concentrations under each condition. In our study, we used parafilm made of polyolefin and paraffin wax, referred to as PP parafilm. Plastic wrap was made of polyethylene, referred to as PE plastic wrap. Surgical tape was made of non-woven fabric and plastic, referred to as NF surgical tape. Our study aimed to (1) examine the impact of commonly used laboratory sealing films on seedling growth, (2) identify specific genes linked to reduced size and delayed growth due to sealing, and (3) raise awareness about the potential influence of sealing on experimental results, ensuring more accurate and reliable outcomes.

## 2. Results

### 2.1. The Growth of Arabidopsis Thaliana Was Affected by Sealing in Petri Dishes

**Figure 2:**
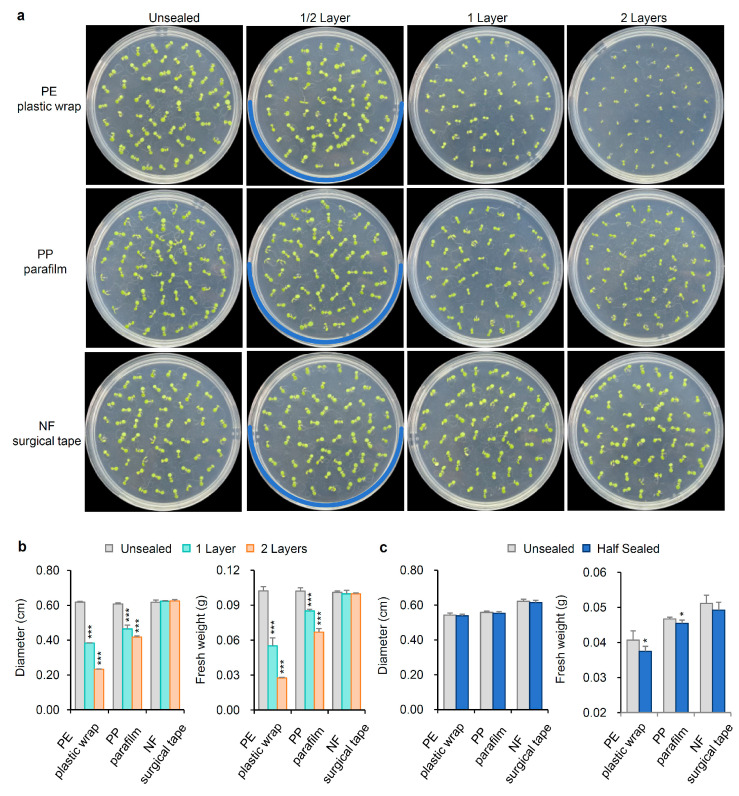
Phenotype of seedlings grown in Petri dishes for 7 days sealed with different films and layers. (**a**) Seedlings in Petri dishes sealed with PE plastic wrap, PP parafilm, and NFsurgical tape 0, ½, 1, and 2 layers. 0 represented the Petri dish was not sealed. The blue curve represents the position of the ½ layer sealing film. (**b**) The diameter and fresh weight of seedlings in Petri dishes sealed with PE plastic wrap, PP parafilm, and NF surgical tape 0, 1, and 2 layers. (**c**) The diameter and fresh weight of seedlings in half-sealed Petri dishes with PE plastic wrap, PP parafilm, and NF surgical tape. The diameter and fresh weight were measured for 50 seedlings from each Petri dish. Measuring the diameter and fresh weight of 25 seedlings in half of the sealed Petri dish. Standard deviation was used to calculate error bars. Statistical significance was based on a Student’s *t*-test (* *p* < 0.05 and *** *p* < 0.001) compared to the unsealed condition. The experiments were repeated three times with similar results.

After 7 days of growth under a 16h light/8h dark cycle at 22 °C, unsealed Arabidopsis seedlings reached a stage with fully extended cotyledons and two visible true leaves. The diameter and fresh weight of unsealed seedlings were 0.61 ± 0.01 cm and 0.10 ± 0.01 g, respectively. In contrast, seedlings in dishes sealed with one or two layers of PE plastic wrap or PP parafilm were less developed, with only cotyledons visible and no distinct true leaves. Seedlings grown in dishes sealed with PE plastic wrap were notably smaller than those in PP parafilm-sealed dishes. Specifically, seedlings in dishes sealed with one and two layers of PE plastic wrap showed reduced diameters of 0.38 ± 0.01 cm and 0.23 ± 0.01 cm, with fresh weights of 0.055 ± 0.01 g and 0.027 ± 0.01 g, respectively. Compared to unsealed conditions, the diameter loss rates were as high as 37.70% and 62.30%, respectively. And the weight loss rates were as high as 45.00% and 73.00%. The loss rate refers to the diameter or fresh weight of plants under unsealed conditions minus the diameter or fresh weight under sealed conditions, and then divided by the diameter or fresh weight under unsealed conditions. Seedlings in dishes sealed with one and two layers of PP parafilm had diameters of 0.46 ± 0.02 cm and 0.42 ± 0.01 cm and fresh weights of 0.085 ± 0.01 g and 0.067 ± 0.01 g, respectively. Correspondingly, the diameter loss rates were 24.59% and 31.15%, and the weight loss rates were 15% and 33%, respectively. In contrast, seedlings in dishes sealed with one or two layers of NF surgical tape maintained a stable diameter and fresh weight (approximately 0.62 ± 0.01 cm and 0.10 ± 0.01 g), comparable to unsealed seedlings (Figure 2a,b). The growth stage and development of seedlings in NF surgical tape-sealed dishes were almost identical to those in unsealed dishes. Additionally, we compared growth within the same Petri dish by sealing only half with PE plastic wrap, PP parafilm, or NF surgical tape. Seedlings in the PE plastic wrap or PP parafilm-sealed halves showed no significant difference in diameter compared to unsealed halves but exhibited a significant reduction in fresh weight, suggesting that root growth was inhibited in the portions of dishes sealed with PE plastic wrap or PP parafilm (Figure 2c).

**Figure 3:**
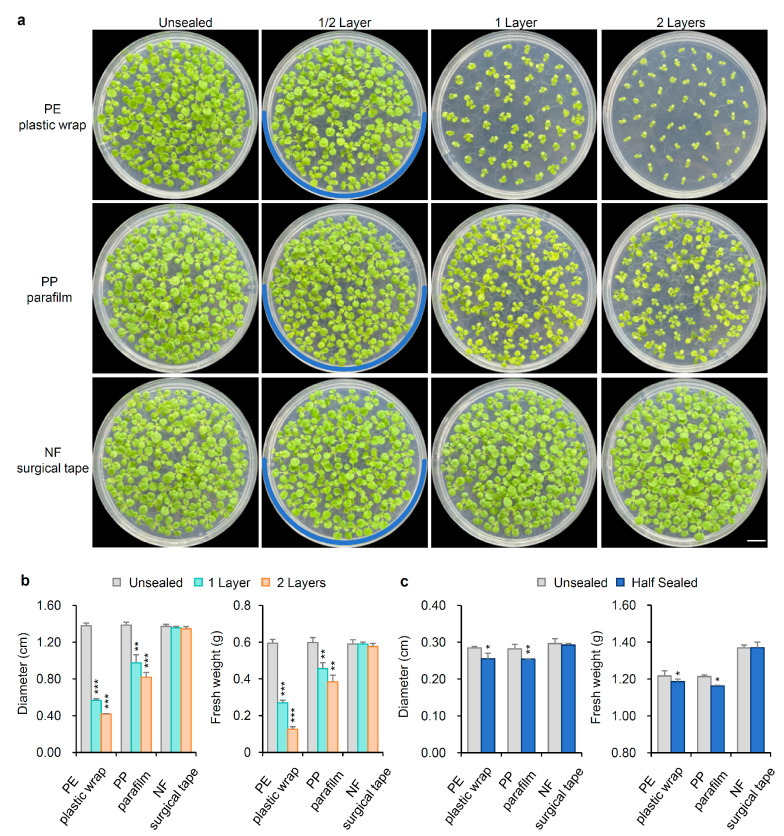
Phenotype of seedlings grown in Petri dishes for 14 days sealed with different films and layers. (**a**) Seedlings in Petri dishes sealed with PE plastic wrap, PP parafilm, and NF surgical tape 0, ½, 1, and 2 layers. 0 represented the Petri dish was not sealed. The blue curve represents the position of the ½ layer sealing film. (**b**) The diameter and fresh weight of seedlings in Petri dishes sealed with PE plastic wrap, PP parafilm, and NF surgical tape 0, 1, and 2 layers. (**c**) The diameter and fresh weight of seedlings in half-sealed Petri dishes with PE plastic wrap, PP parafilm, and NF surgical tape. The diameter and fresh weight were measured for 50 seedlings from each Petri dish. Measuring the diameter and fresh weight of 25 seedlings in half of the sealed Petri dish. Standard deviation was used to calculate error bars. Statistical significance was based on a Student’s *t*-test (* *p* < 0.05, ** *p* < 0.01, and *** *p* < 0.001) compared to the unsealed condition. The experiments were repeated three times with similar results.

For 14-day-old seedlings, unsealed plants displayed the third pair of true leaves, with minimal size variation among individuals. The diameter and fresh weight of unsealed seedlings increased to 1.38 ± 0.03 cm and 0.59 ± 0.02 g, respectively, comparable to those of seedlings in Petri dishes sealed with NF surgical tape, which measured 1.36 ± 0.02 cm in diameter and 0.59 ± 0.01 g in fresh weight. In contrast, seedlings in dishes sealed with a single layer of PE plastic wrap or PP parafilm were at the second true leaf stage, with plants in PE plastic wrap-sealed dishes showing smaller growth. The diameter and fresh weight of seedlings grown in dishes sealed with one layer of PE plastic wrap were 0.57 ± 0.01 cm and 0.27 ± 0.01 g, respectively, while those in PP parafilm-sealed dishes measured 0.98 ± 0.08 cm and 0.46 ± 0.03 g. Hence, in dishes sealed with one layer of PE plastic wrap and PP parafilm, the diameter loss rates were 58.70% and 28.99%, and the weight loss rates were 54.24% and 22.03%, respectively. In dishes sealed with two layers of PE plastic wrap, seedling development was notably delayed, showing only the first pair of true leaves. Consequently, diameter and fresh weight were reduced to 0.42 ± 0.01 cm and 0.13 ± 0.01 g, respectively. For seedlings in dishes sealed with two layers of PP parafilm, these measurements were 0.82 ± 0.05 cm in diameter and 0.38 ± 0.03 g in fresh weight (Figure 3a,b). Therefore, in dishes sealed with two layers of PE plastic wrap and PP parafilm, the diameter loss rates were as high as 69.57% and 40.58%, and the weight loss rates were as high as 77.97% and 35.59%, respectively. In half-sealed Petri dishes, seedlings in PE plastic wrap- and PP parafilm-sealed sections exhibited smaller diameters and lower fresh weights than those in unsealed sections. Conversely, in dishes half-sealed with NF surgical tape, there were no significant differences in size and weight between the sealed and unsealed halves (Figure 3c).

In summary, both PE plastic wrap and PP parafilm sealing compromised plant growth, whereas NF surgical tape did not. Additionally, seedlings in PE plastic wrap-sealed dishes were smaller than those in PP parafilm-sealed dishes. For any given sealing type, increased layers around the Petri dish further reduced seedling growth.

### 2.2. Transcriptome Analysis

To investigate the cause of compromised growth in sealed Petri dishes, we performed RNA-seq analysis on *Arabidopsis thaliana* seedlings grown in dishes sealed with one layer of PE plastic wrap (abbreviated as PW), PP parafilm (abbreviated as PF), and NF surgical tape (abbreviated as ST) for 7 and 14 days (7 d and 14 d). Unsealed Petri dishes served as controls (CK7d and CK14d) for each time point.

A total of 24 RNA samples were collected from CK7d, PW7d, PF7d, ST7d, CK14d, PW14d, PF14d, and ST14d, with three biological replicates per condition. RNA-seq generated 1077.07 million clean reads, with each sample ranging from 44.67 to 45.09 million reads, Q20 values exceeding 98.59%, and Q30 values over 94.72%. The alignment of clean reads to the *Arabidopsis* reference genome (TAIR10) showed an average alignment ratio of 98.30%, with 96.34% of reads uniquely mapped. The number of expressed genes across all samples was similar, ranging from 18,335 to 19,213 (Table S1).

**Figure 4:**
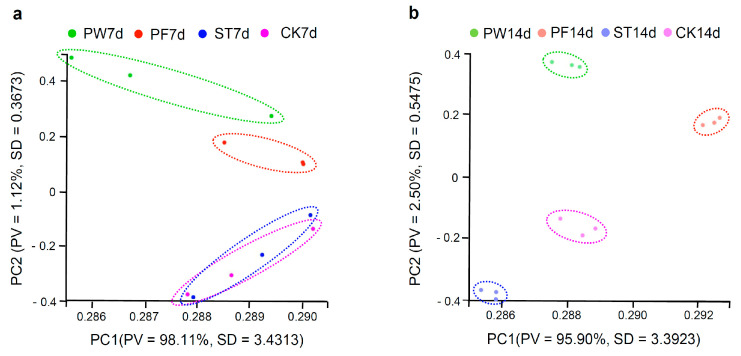
Principal component analysis (PCA). (**a**) A 7-day-old seedlings in Petri dish sealed with PE plastic wrap (PW7d), PP parafilm (PF7d), and NF surgical tape (ST7d) with twelve cDNA libraries. (**b**) A 14-day-old seedlings in Petri dish sealed with PE plastic wrap (PW14d), PP parafilm (PF14d), and NF surgical tape (ST14d) with twelve cDNA libraries. PV represents proportion of variance, and SD represents standard deviation. One point represents one sample, and samples from the same group are represented by the same color.

Principal component analysis (PCA) was performed based on expressed genes, separately analyzing the 7-day and 14-day seedling groups. For 7-day-old seedlings (Figure 4a), samples from PE plastic wrap-sealed (PW7d) and PP parafilm-sealed (PF7d) dishes formed distinct clusters, while samples from NF surgical tape-sealed dishes (ST7d) overlapped with those from unsealed dishes (CK7d), suggesting substantial overlap in gene expression between ST7d and CK7d. Unsealed samples (CK7d) were clearly separated from those grown in PE plastic wrap- and PP parafilm-sealed dishes. PCA for 14-day-old seedlings (Figure 4b) showed distinct clustering of plants from each condition—unsealed (CK14d);PE plastic wrap-sealed (PW14d);PP parafilm-sealed (PF14d); and NF surgical tape-sealed (ST14d)—indicatinga large number of DEGs between sealed and unsealed conditions.

### 2.3. DEG Analysis

For 7-day-old seedlings, RNA-seq analysis identified 1814 DEGs between PE plastic wrap-sealed and unsealed seedlings (CK7d vs. PW7d), with 643 upregulated and 1171 downregulated genes. In PP parafilm-sealed versus unsealed seedlings (CK7d vs. PF7d), a total of 1083 DEGs were detected, including 635 upregulated and 448 downregulated genes. By contrast, only two upregulated DEGs were found between NF surgical tape-sealed and unsealed seedlings (CK7d vs. ST7d). Thus, different sealing materials led to varying numbers of DEGs, with PE plastic wrap causing the most, followed by PP parafilm, and then NF surgical tape. This aligns with observed phenotypes: Seedlings in PE plastic wrap-sealed dishes exhibited the smallest growth, followed by those in PP parafilm-sealed dishes, while growth in NF surgical tape-sealed dishes was nearly equivalent to unsealed conditions.

At 14 days, the number of DEGs increased under each sealing condition compared to 7 days. In PE plastic wrap-sealed versus unsealed seedlings (CK14d vs. PW14d) and PP parafilm-sealed versus unsealed seedlings (CK14d vs. PF14d), 2644 and 2410 DEGs were identified, respectively. Consistent with these gene expression changes, seedlings in PE plastic wrap- and PP parafilm-sealed dishes exhibited notably smaller sizes compared to unsealed seedlings. Interestingly, although NF surgical tape sealing did not visibly alter growth compared to unsealed dishes, it resulted in differential expression of 2246 genes, with 751 upregulated and 1495 downregulated genes (CK14d vs. ST14d) (Table 1).

### 2.4. Enriched Biological Process of DEGs

**Figure 5:**
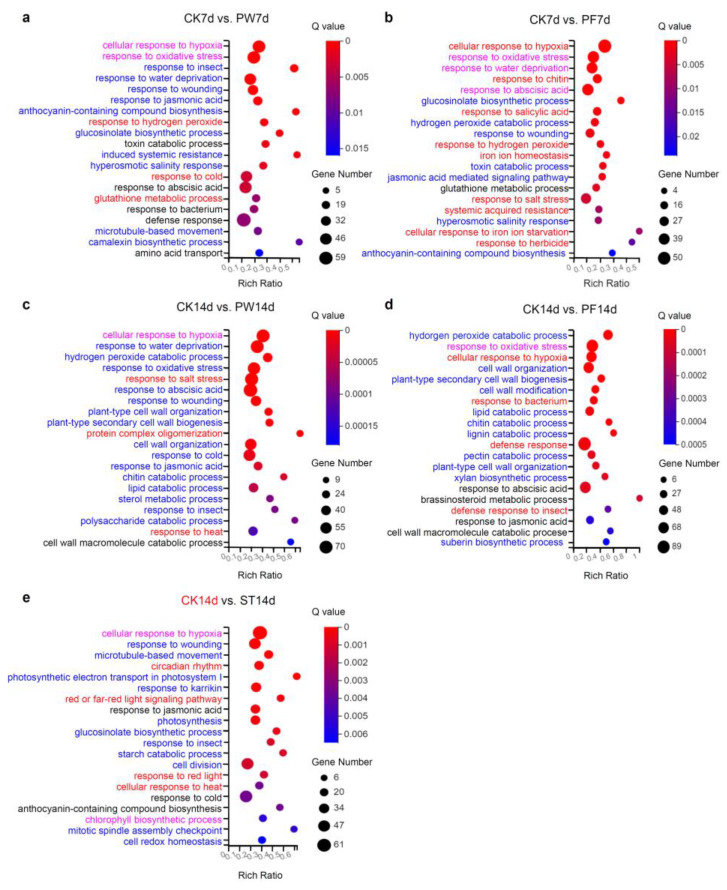
Gene ontology (GO) analysis. (**a**,**b**) Biological process annotation of differentially expressed genes (DEGs) between 7-day-old seedlings sealed with PE plastic wrap, PP parafilm, and those not sealed (CK7d vs. PW7d and CK7d vs. PF7d). (**c**–**e**) The top 20 biological process enrichments of DEGs resulting from 14-day-old seedlings in Petri dishes sealed with PE plastic wrap, PP parafilm, and NF surgical tape compared to those in unsealed Petri dishes (CK14d vs. PW14d, CK14d vs. PF14d, and CK14d vs. ST14d). The *x*-axis represented the enrichment ratio, and the *y*-axis represented the GO term. The magenta font represented the GO term, which appeared in both the top 20 biological processes of upregulated DEG enrichment and the top 20 biological processes of downregulated DEG enrichment. The GO terms displayed in red and blue font represent their occurrence only in the top 20 biological processes enriched in upregulated DEGs and downregulated DEGs, respectively. The GO terms displayed in black font represent these terms that did not appear in the top 20 enrichment of upregulated DEGs nor in the top 20 enrichment of downregulated DEGs. The size of the bubble represented the number of genes annotated to a certain GO term. The color represents the significant value of enrichment. A Q value less than 0.05 is used as a criterion for determining significance.

To investigate the functional pathways affected by different sealing films, we analyzed DEGs associated with each sealing condition in terms of GO biological processes (BPs). The first 20 enriched BPs were displayed (Figure 5).

At the 7-day mark, the top five significantly enriched BPs among DEGs in seedlings sealed with PE plastic wrap were a cellular response to hypoxia, response to oxidative stress, response to insects, response to water deprivation, and response to wounding (Figure 5a, Supplementary Data Set 2, Sheet 1-1). Further analysis revealed that upregulated genes were primarily associated with processes such as response to hydrogen peroxide, response to cold, and glutathione metabolic process (red fonts in Figure 5a, Supplementary Data Set 2, Sheet 1-2). In contrast, downregulated genes were enriched in processes such as response to insects, response to water deprivation, response to wounding, response to jasmonic acid, and anthocyanin-containing compound biosynthetic process (blue fonts in Figure 5a, Supplementary Data Set 2, Sheet 1-3). In addition, both upregulated genes and downregulated genes were enriched in cellular response to hypoxia and response to oxidative stress (magenta fonts in Figure 5a, Supplementary Data Set 2 Sheet 1-2, 1-3).

For PP parafilm-sealed seedlings, the most significantly enriched BPs were cellular response to hypoxia, response to oxidative stress, response to water deprivation, response to chitin, and response to abscisic acid (Figure 5b, Supplementary Data Set 2 Sheet 2-1). Upregulated genes were enriched in processes including cellular response to hypoxia, response to chitin, response to salicylic acid, response to hydrogen peroxide, and iron ion homeostasis (red fonts in Figure 5b, Supplementary Data Set 2 Sheet 2-2). Downregulated genes were associated with the glucosinolate biosynthetic process, hydrogen peroxide catabolic process, response to wounding, toxin catabolic process, jasmonic acid-mediated signaling pathway, and anthocyanin-containing compound biosynthetic process (blue fonts in Figure 5b, Supplementary Data Set 2, Sheet 2-3). Upregulated genes and downregulated genes were enriched in common processes including response to oxidative stress, response to water deprivation, and response to abscisic acid (magenta fonts in Figure 5b, Supplementary Data Set 2 Sheet 2-2, 2-3). The two DEGs identified in NF surgical tape-sealed seedlings did not show significant enrichment in any BP.

For 14-day-old seedlings, PE plastic wrap-sealed conditions resulted in DEGs primarily enriched in cellular response to hypoxia, response to water deprivation, hydrogen peroxide catabolic process, response to oxidative stress, and response to salt stress. Among these, response to water deprivation, hydrogen peroxide catabolic process, and response to oxidative stress were enriched in downregulated DEGs, while cellular response to hypoxia was enriched in both upregulated and downregulated DEGs (Figure 5c, Supplementary Data Set 2, Sheet 3-1, 3-2, 3-3).

In PP parafilm-sealed dishes, the top five enriched BPs included hydrogen peroxide catabolic process, response to oxidative stress, cellular response to hypoxia, cell wall organization, and plant-type secondary cell wall biogenesis. Here, cellular response to hypoxia was enriched in upregulated DEGs (red fonts in Figure 5d, Supplementary Data Set 2 Sheet 4-2). Response to oxidative stress was enriched in both upregulated and downregulated DEGs (magenta in Figure 5d, Supplementary Data Set 2 Sheet 4-2, 4-3). While the other three categories were enriched in downregulated DEGs (Figure 5d, Supplementary Data Set 2 Sheet 4-3).

For NF surgical tape-sealed conditions, DEGs were enriched in BPs such as cellular response to hypoxia, response to wounding, microtubule-based movement, circadian rhythm, and photosynthetic electron transport in photosystem I. The circadian rhythm process was enriched in upregulated DEGs, while response to wounding, microtubule-based movement, and photosynthetic electron transport in photosystem I were enriched in downregulated DEGs. Cellular response to hypoxia appeared in both upregulated and downregulated gene enrichment (Figure 5e, Supplementary Data Set 2, Sheet 5-1, 5-2, 5-3).

In summary, cellular response to hypoxia was a significantly enriched biological process across all sealing types—PE plastic wrap;PP parafilm; and NF surgical tape. Additionally, cellular response to hypoxia and response to oxidative stress were common enriched processes resulting from PE plastic wrap and PP parafilm sealing in both 7- and 14-day-old seedlings.

It was noticed that some categories, such as “cellular response to hypoxia” in groups “CK7d vs. PF7d” and “CK14d vs. PF14d”, only appeared in upregulated gene enrichment (red font in Figure 5b,d and Supplementary Data Set 2, Sheet 2-2, and 4-2). But cellular response to hypoxia was enriched in both upregulated and downregulated DEGs in groups “CK7d vs. PW7d”, “CK14d vs. PW14d”, and “CK14d vs. ST14d” (magenta font in Figure 5a,c,e and Supplementary Data Set 2, Sheet 1-2, 1-3, 3-2, 3-3, 5-2, and 5-3). Response to oxidative stress was enriched in downregulated DEGs in the group “CK14d vs. PW14d” (blue font in Figure 5c), while in groups “CK7d vs. PW7d”, “CK7d vs. PF7d”, and “CK14d vs. PF14d”, it appeared in both upregulated and downregulated gene enrichment (magenta font in Figure 5a,b,d).

GSEA (Gene Set Enrichment Analysis) displayed that “cellular response to hypoxia” was significantly enriched in “CK7d vs. PF7d”, and “CK14d vs. PF14d” with nominal *p*-values < 0.05, false discovery rate (FDR) < 0.25, and |normalized enrichment score (NES)| > 1 (Figure S1b,d). In addition, due to ES > 0, it suggested that the “cellular response to hypoxia” pathway was upregulated, which was consistent with the conclusion of GO analysis (Figure 5b,d). However, “cellular response to hypoxia” in the other three groups of “CK7d vs. PW7d”, “CK14d vs. PW14d”, and “CK14d vs. ST14d”, with nominal *p*-values > 0.05, indicated that the genes were randomly distributed (Figure S1a,c,e).

### 2.5. Different Sealing Methods Affect CO_2_ but Not O_2_ Content in Petri Dishes

**Figure 6:**
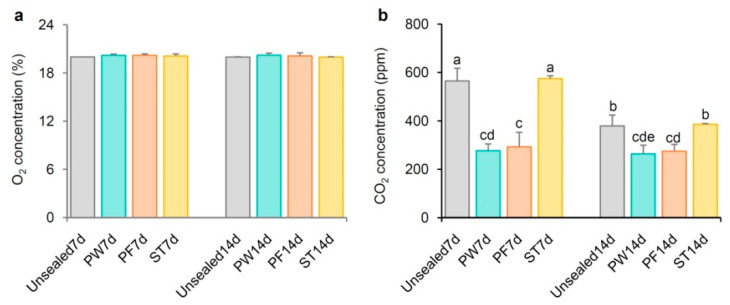
Concentration of CO_2_ and O_2_ in Petri dishes where seedlings grew for 7 and 14 days. (**a**) O_2_ concentration in Petri dishes. (**b**) CO_2_ concentration in dishes. A 7d and 14d represented 7- and 14-day-old seedlings in Petri dishes. Standard deviation was used to calculate error bars. “a” to “e” referred to two-way ANOVA tests. The experiment was repeated at least three times, and one representative experimental result was exhibited.

Given the observed enrichment of cellular response to hypoxia in seedlings sealed with PE plastic wrap, PP parafilm, and NF surgical tape, we investigated the O_2_ and CO_2_ levels inside Petri dishes sealed with each type of film. Due to staggered sowing, we can measure seedlings that have grown for 7 days or 14 days on the same day. We found that regardless of whether seedlings were 7 or 14 days old, no significant differences in O_2_ content were found among dishes sealed with PE plastic wrap, PP parafilm, or NF surgical tape compared to unsealed dishes (Figure 6a). However, CO_2_ levels were significantly lower in dishes sealed with PE plastic wrap and PP parafilm, while no significant decrease in CO_2_ content was observed in dishes sealed with NF surgical tape. In addition, compared to seedlings that have grown for 7 days, 14-day-old seedlings have lower CO_2_ concentrations in their Petri dishes (Figure 6b).

### 2.6. Identification of Responsive Genes for Compromised Growth

**Figure 7:**
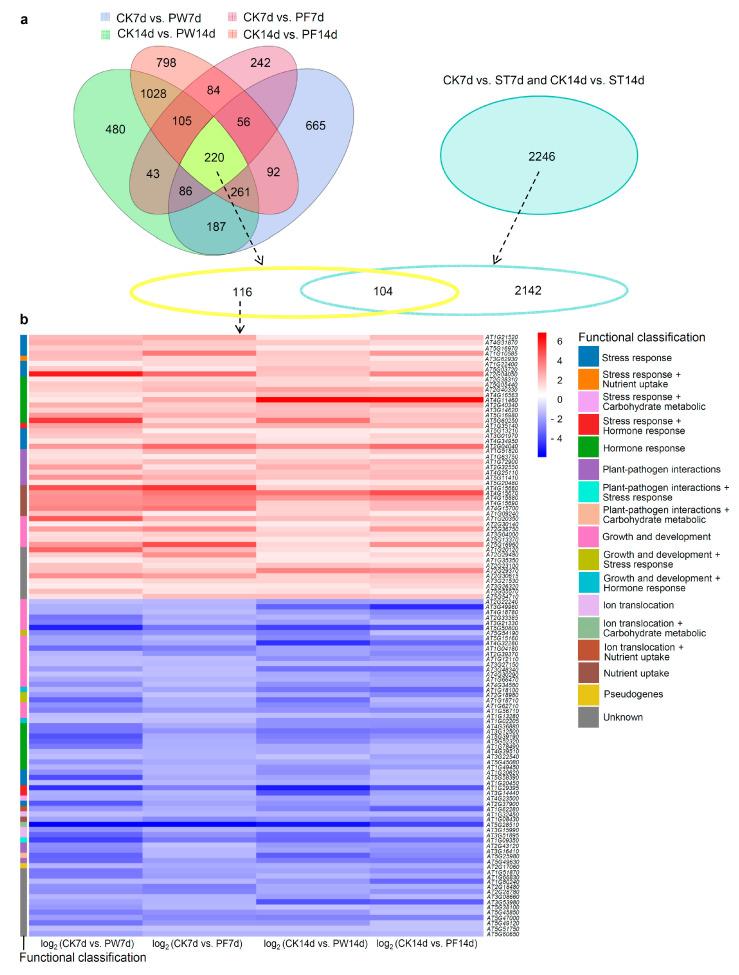
Identification of responsive genes related to compromised growth. (**a**) Venn diagrams showed 220 common DEGs among CK7d vs. PW7d, CK7d vs. PF7d, CK14d vs. PW14d, and CK14d vs. PF14d. Subtracting the DEGs caused by NF surgical tape resulted in 116 DEGs. (**b**) Heatmap and functional classification of 116 responsive genes related to compromised growth.

Both PE plastic wrap and PP parafilm sealing led to delayed growth phenotypes in *Arabidopsis* seedlings. The analysis identified 609 common DEGs from PE plastic wrap and PP parafilm-sealed samples at 7 days (CK7d vs. PW7d and CK7d vs. PF7d) and 1614 common DEGs at 14 days (CK14d vs. PW14d and CK14d vs. PF14d), resulting in 220 overlapping DEGs across all conditions (Table 1, Figure 7a). NF surgical tape sealing also produced 2246 DEGs over 7 and 14 days (Table 1, Figure 7a), yet did not visibly affect growth or development. By excluding DEGs unique to NF surgical tape, we identified 116 genes that may be related to compromised growth specifically under PE plastic wrap and PP parafilm conditions. These included 51 upregulated and 65 downregulated genes (Figure 7a,b).

Among the upregulated genes, several were responsive to oxidative stress, including *AT1G21520*, *AT4G31870 (ATGPX7)*, *AT5G16970* (*AER*), *AT1G10585,* and *AT3G62930* (*ROXY17*). Several were responsive to heat or salt stress, such as *AT1G22400* (*UGT85A1*), *AT5G03720* (*HSFA3*), and *AT2G04050*. Additionally, some genes were associated with stress hormone responses, such as *AT2G38310* (*PYL4*), *AT5G05440* (*PYL5*), *AT2G40330* (*PYL6*), *AT4G16563*, and *AT4G11460* (*CRK30*), which participate in abscisic acid (ABA) signaling, and *AT2G40340* (*DREB2C*), known to modulate ABA biosynthesis. PYL4, PYL5, and PYL6 encode members of the PYR/PYL/RCAR family, functioning as ABA receptors. Stress-related genes *AT3G14620* (*CYP72A8*) and *AT5G16980* responded to xenobiotic stress, while *AT5G60250* was linked to cytokinin response. Other upregulated genes were responsive to nutrient starvation or heavy metal stress. These included *AT1G35140 (EXL1)*, *AT5G13210*, *AT3G01970* (*WRKY45*), *AT4G34950* (*MFS1*), and *AT2G04040* (*ATDTX1*). Immune-related genes included *AT1G51820* (*SIF4*), *AT1G63750*, *AT1G72900* (*TN7*), *AT2G32550* (*NOT9C*), *AT4G25110* (*AtMC2*), *AT5G11410* (*SZE2*), and *AT5G20480* (*EFR*). Additionally, six genes associated with nutrient uptake and six genes involved in growth and development were upregulated (Table 2) [6,7,8,9,10,11,12,13,14,15,16,17,18,19,20,21,22,23,24,25,26,27,28,29,30,31,32,33,34,35,36,37,38,39,40,41,42,43,44,45,46,47,48,49,50,51,52,53,54,55,56].

Among the 65 downregulated genes, after excluding 13 genes with unknown functions and one pseudogene, 51 remained. Of these, 24 were associated with growth and development, accounting for nearly half of the downregulated genes. For example, *AT2G22240* (*ATMIPS2*), *AT3G49960 (PRX35), AT4G18780* (*CESA8/IRX1*), *AT2G33385 (ARPC2B)*, *AT3G21330*, *AT5G50800* (*SWEET13*), and *AT5G54190* (*PORA*) were essential for proper seedling growth and development. Genes such as *AT5G15160* (*BNQ2*), *AT4G32280* (*IAA29*), *AT1G04180* (*YUC9*), *AT1G12110* (*CHL1*), *AT2G39370* (*MAKR4*)*, AT3G27150* (*HOLT*), *AT4G30290* (*XTH19*), and *AT3G48340* (*CEP2*) contribute to hypocotyl and/or root/lateral root growth, while *AT1G66470* (*RHD6*) and *AT4G34580* (*COW1*) function in root hair initiation and growth. Additionally, *AT1G18100* (*MFT*), *AT2G18980* (*PRX16*), *AT1G18710* (*ATMYB47*), and *AT1G62710* (*AtAEP2; β-VPE*) were involved in seed germination, longevity, and protein maturation. *AT1G56710* (*PGL1*), *AT1G13280* (*AOC4*), and *AT1G02205* (*CER1*) were related to fertility. Several genes were related to hormone responses, including *AT4G36880* (*AtCP1*) in gibberellin signaling; *AT3G12500* (*ATHCHIB*), *AT5G39190* (*GER2*), and *AT5G52320* (*CYP96A4*) in ethylene/jasmonic acid pathways; *AT1G78490* (*CYP708A3*) and *AT4G39510* (*CYP96A12*) in brassinolide response; *AT3G22540* and *AT5G45080 (PP2-A6)* in cytokinin response; and *AT1G18100*, *AT1G02205*, *AT1G49450* (*AIW2*), *AT1G29395* (*COR413IM1*), and *AT3G14440* (*ATNCED3*) in ABA response. Furthermore, *AT1G18100* and *AT1G02205* were involved in growth and development, while *AT1G29395* and *AT3G14440* were also involved in stress response. Several additional genes were linked to abiotic stress responses, such as *AT1G20620* (*ATCAT3*) and *AT5G38390* (*AtP44*) in oxidative stress and *AT1G20450* (*ERD10*), *AT2G37900*, and *AT4G23500* (*PGF12)* in high salinity, drought, cold, or heavy metal stress. Six additional genes were associated with ion homeostasis or nutrient uptake. The remaining five genes were involved in immune responses (Table 3) [27,57,58,59,60,61,62,63,64,65,66,67,68,69,70,71,72,73,74,75,76,77,78,79,80,81,82,83,84,85,86,87,88,89,90,91,92,93,94,95,96,97,98,99,100,101,102,103,104,105,106,107,108,109,110,111,112,113,114,115,116,117,118,119,120,121,122,123,124,125,126,127,128,129,130,131,132,133,134,135,136,137,138,139,140].

In summary, stress-related genes were predominant among upregulated genes, while growth- and development-related genes were predominant among downregulated ones. This gene expression profile aligns with the delayed and compromised growth phenotype observed in PE plastic wrap- and PP parafilm-sealed Petri dishes, likely due to the stress-inducing environment created by these sealing methods.

### 2.7. Transcriptome Data Validated by qRT-PCR

**Figure 8:**
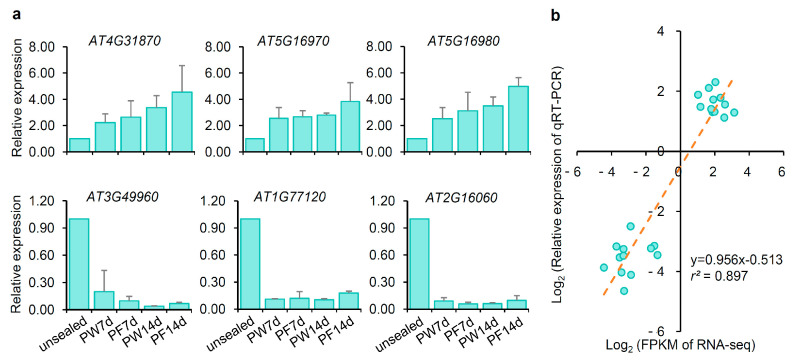
Transcriptional level analysis of some DEGs. (**a**) qRT-PCR detection relative expression levels of *AT4G31870*, *AT5G16970*, *AT5G16980*, *AT3G49960*, *AT1G77120*, and *AT2G16060*. (**b**) Correlation analysis between relative expression of qRT-PCR and RNA-Seq data. Each dot in the scatter chart represented a data point whose position was the corresponding value of the data point on the *x*-axis and *y*-axis. The dash line represented the trend line. The experiments were repeated twice. Mean represented the average value of two biological replicate experiments. Values were expressed as mean ± SD (standard deviation).

To confirm the reliability of the transcriptome data, we selected genes potentially involved in oxidative stress or hypoxia signaling for validation through qRT-PCR. Among these genes, *AT4G31870* (*ATGPX7*), a chloroplast-localized peroxidase involved in the breakdown of H_2_O_2_ to water [8], was upregulated in PE plastic wrap- and PP parafilm-sealed Petri dishes (Figure 8a, Table 2). Conversely, *AT3G49960* (*PRX35*), a peroxidase gene primarily associated with plant developmental processes, was downregulated (Figure 8a, Table 3) [58]. *AT5G16970*, encoding 2-alkenal reductase, which scavenges ROS-derived carbonyls to enhance stress tolerance [11,12], was upregulated under PE plastic wrap- and PP parafilm-sealed conditions (Figure 8a, Table 2). Similarly, *AT5G16980*, a gene induced by phytoprostanes (which inhibit cell division and root growth) [26], was also upregulated, consistent with the observed delayed growth and reduced seedling size in membrane-sealed dishes (Figure 8a, Table 2). We further examined two genes involved in low-oxygen signaling, *AT1G77120* (*ADH1*) and *AT2G16060* (*HB1*). *ADH1*, an alcohol dehydrogenase, and *HB1*, a class 1 nonsymbiotic hemoglobin, are both known to be induced by low oxygen levels [141,142]. However, both genes were downregulated under film-sealed conditions. Notably, *ADH1* and *HB1* appeared in the DEG list for 14-day samples sealed with NF surgical tape and were therefore excluded from Table 3. Additionally, qRT-PCR expression levels showed a strong linear correlation with transcriptome data under PE plastic wrap- and PP parafilm-sealed conditions at 7 and 14 days (y = 0.956x − 0.513, *r*^2^ = 0.897, *p* = 2.34 × 10^−12^) (Figure 8b), confirming the reliability of the transcriptome results.

## 3. Discussion

We unexpectedly observed that *Arabidopsis* plants grown in film-sealed Petri dishes exhibited altered vegetative phenotypes, including reduced size and delayed growth. It is well known that plant growth can slow or even halt under environmental stress conditions, often resulting in decreased cell numbers, reduced mitotic activity, and slower cell division rates [143,144]. This reduction in cell division under stress likely conserves energy and minimizes the risk of heritable damage [145,146]. Genetically, plants undergo extensive reprogramming to adapt to adverse environmental conditions [147,148,149].

To understand the causes of the reduced size and delayed growth observed in film-sealed dishes, we conducted RNA-seq analysis. Our findings revealed that PE plastic wrap- and PP parafilm-sealed dishes significantly enriched DEGs related to “hypoxia response” and “oxidative stress” on both day 7 and day 14.

Although there were inconsistencies in the results from GO and GSEA analysis for “cellular response to hypoxia” in groups “CK7d vs. PW7d”, “CK14d vs. PW14d”, and “CK14d vs. ST14d”, this contradiction is understandable as it may be due to the following reasons. Firstly, “cellular response to hypoxia”, which appeared in the enrichment BPs of both upregulated and downregulated DEGs in “CK7d vs. PW7d”, “CK14d vs. PW14d”, and “CK14d vs. ST14d”. It is possible that the expression levels of upregulated and downregulated genes compensate for each other, resulting in this gene set appearing insignificant in GSEA. Secondly, the GO analysis evaluates the functional enrichment of genes by comparing the proportion of upregulated or downregulated genes in the pathway and whether it is significantly higher than the proportion of background genes in this pathway. It is an analysis using differentially expressed genes [150]. It is suitable for mining individual genes that have large effects on the phenotype. GSEA appears to have greater power to detect small but biologically important changes in a set of genes. It uses all expressed genes for analysis. It is particularly suitable for mining gene sets that many genes each make subtle contributions to [151]. In addition, there are significant differences in algorithms between GO and GSEA. Hence, the significant enrichment pathways they found may be different.

Measurements of O_2_ and CO_2_ levels indicated a significant decrease in CO_2_ concentration in PE plastic wrap- and PP parafilm-sealed dishes compared to unsealed conditions, with no significant change in O_2_ levels. This suggests that while the sealing did not lead to an O_2_ deficiency, it did reduce CO_2_ levels in the sealed Petri dishes.

It is well established that Rubisco (ribulose-1,5-bisphosphate carboxylase/oxygenase) catalyzes the carboxylation of RuBP (ribulose-1,5-bisphosphate), producing two molecules of 3-phosphoglycerate (3-PGA) per CO_2_ fixed. Then, 3-PGA enters the Calvin-Benson cycle (CBBC), where it is reduced to triose phosphates, which serve as precursors for nearly all essential compounds in the plant [152]. Under low CO_2_/O_2_ conditions, however, Rubisco favors its oxygenase function, leading to the production of high levels of 2-phosphoglycolate (2-PG) [153]. This compound inhibits photosynthetic carbon fixation, representing a loss of carbon and disrupting carbon allocation [154,155]. Consequently, a low CO_2_ environment hinders starch accumulation and alters soluble sugar levels. Such a low CO_2_/O_2_ environment parallels the conditions inside Petri dishes sealed with PE plastic wrap or PP parafilm, resulting in the downregulation of genes like *AT4G23500*, *AT5G28510*, and *AT5G25980*, which are involved in carbohydrate metabolism and contribute to the observed growth limitations in seedlings (Table 3) [112,119,127,128].

When carbon and sucrose supply is poor or low CO_2_ environment, the expression level of *AT1G35140* (*EXL1*) and *AT5G13210* increases [28,29,30]. Previous studies revealed that nitrate-upregulated glutaredoxins—AtGRXS3; *AtGRXS4; AtGRXS5*; and *AtGRXS7*—were negative regulators of primary root growth [42]. Under film-sealed conditions, the expression of these genes is upregulated (Table 2). Nutrient uptake and homeostasis are crucial for maintaining normal plant growth. Multiple genes involved in ion transport appear in the downregulated gene list, suggesting that ion translocation is hindered under film-sealed conditions, leading to plant growth inhibition.

Carbohydrates play a crucial role in plant-pathogen interactions, and reductions in carbohydrate levels may drive the expression of genes involved in pathogen response [123]. Accordingly, both the upregulated and downregulated gene lists associated with compromised growth contain multiple genes involved in plant-pathogen interactions.

The reduction in CO_2_ levels not only impacts carbohydrate accumulation but also intensifies photorespiration and disrupts the redox balance within plants. In previous studies, the *cat2* photorespiration mutant was screened by sealing Petri dishes with multiple layers of PP parafilm to limit air exchange, causing a rapid CO_2_ decrease and increased flux through the photorespiratory pathway [156]. Similarly, soybean (*Glycine max*) photorespiration mutants were screened under low CO_2_ conditions [157]. Plant growth and development are highly dependent on maintaining redox homeostasis [158,159,160,161,162], with H_2_O_2_ levels serving as a key indicator of the cellular redox state [163]. When H_2_O_2_ levels reach high concentrations, they exert toxic effects on the plant. Peroxidases and catalases function as ROS scavengers to mitigate high H_2_O_2_ levels via reduction (peroxidases) or dismutation (catalases) [156]. Our results revealed that the peroxidase *AT4G31870* (*ATGPX7*) was upregulated (Table 2) [8,9,10], which parallels the significant accumulation of *ATGPX7* observed in the *cat2* mutant under photorespiration-promoting conditions [10]. In contrast, catalase (*AT1G20620*) and another peroxidase (*AT5G58390*) were downregulated (Table 3) [106,107,109]. Despite robust antioxidant mechanisms, environmental stress can still disrupt redox balance, and the fate of cells is influenced by the equilibrium between ROS production and clearance. Under film-sealed conditions, the upregulation and downregulation of multiple peroxidase and catalase genes suggest an active attempt by plants to balance ROS levels in response to stress.

Moreover, mutants of the PYR/RCAR abscisic acid (ABA) receptor family show impaired CO_2_ signaling [164], suggesting a convergence between CO_2_ and ABA signaling pathways. Among the genes associated with compromised seedling growth, multiple PYR/PYL/RCAR family members, including *PYL4*, *PYL5*, and *PYL6*, were upregulated [21,22]. This indicates an enhanced ABA signaling response under film-sealed, low-CO_2_ conditions.

The core components of hypoxia signal perception are group VII ethylene response factors (ERFs), which are targeted for proteasomal degradation in the presence of oxygen through the N-end rule pathway. Under low O_2_ conditions, stabilization of group VII ERF proteins drives the downstream activation of anaerobic genes essential for anaerobic metabolism [165,166]. Key components of O_2_ perception, RAP2.2 and RAP2.12, two members of the group VII ERFs [142], did not appear in the list of DEGs across all sealing conditions compared to the unsealed condition (Supplementary Data Set 2). Furthermore, the gene lists associated with delayed growth did not include hypoxia-signaling genes. Further analysis revealed that low-oxygen signaling genes such as *HRE2* (*AT2G47520*, another group VII ERF member [165]) and *HRU1* (*AT3G03270*, hypoxia-responsive universal stress protein 1 [167]), both typically induced under low O_2_, were downregulated in 14-day-old seedlings grown in PE plastic wrap-, PP parafilm-, and NF surgical tape-sealed Petri dishes. Additional low O_2_ regulatory components—*PCO* (*AT5G39890*, plant cysteine oxidase controlling the stability of group VII ERFs [168]), *HRA1* (*AT3G10040*, hypoxia response attenuator 1 counteracting *RAP2.12* [169]), and *WRKY70* (*AT3G56400*, interacting with acyl-CoA-binding proteins for hypoxia response [5])—were similarly downregulated under these conditions. Notably, *HRA1* and *WRKY70* were also downregulated in PE plastic wrap-sealed dishes at 7 days. Core hypoxia-responsive genes, including *HB1* (*AT2G16060*, non-symbiotic hemoglobin 1 activated by *RAP2.2*, *RAP2.3*, and *RAP2.12*) and *ADH1* (*AT1G77120*, alcohol dehydrogenase induced by hypoxia), were downregulated in 7-day PE plastic wrap- and PP parafilm-sealed dishes as well as in 14-day PE plastic wrap-, PP parafilm-, and NF surgical tape-sealed dishes [141,170]. These findings suggest that sealing Petri dishes does not activate hypoxia signaling pathway genes and thus does not create an oxygen-deficient environment, consistent with the measured O_2_ levels. The downregulation of hypoxia-responsive genes may reflect inhibited protein synthesis due to compromised growth under film-sealed conditions, as protein synthesis inhibitors are known to impair hypoxia adaptation in *Arabidopsis* roots and stems, leading to reduced expression of hypoxia-induced genes [171].

From a growth and development perspective, the reduced size of seedlings under PE plastic wrap and PP parafilm sealing indicates inhibited plant growth, likely due to suppressed cell division and expansion. During these processes, primary cell walls form to determine cell shape and size, and as plants mature, some cells develop lignified secondary cell walls [172]. The primary components of cell walls are cellulose, hemicellulose, and pectin [173]. Genes involved in cellulose synthesis, including *AT4G18780 (CESA8)*, *AT5G17420 (CESA7)*, and *AT5G44030 (CESA4)* [174], were downregulated (Supplementary Data Set 2). Since cellulose serves as a probable target for α-expansin [175], multiple α-expansin genes, such as *AT1G12560 (EXPA7)*, *AT1G62980 (EXPA18)*, and *AT2G40610 (EXPA8)*, were also downregulated [176,177,178]. Xylan, the most abundant hemicellulose, is synthesized by genes such as *AT3G50220 (IRX15)*, *AT5G67210 (IRX15-L)*, and *AT2G37090 (IRX9)* [179,180,181], all of which showed downregulation. Furthermore, several xyloglucan endotransglucosylase genes, including *AT4G30290 (XTH19)*, *AT5G57530 (XTH12)*, *AT5G57540 (XTH13)*, *AT1G65310 (XTH17)*, and *AT2G14620 (XTH10)*, which reduce cell wall stiffness and promote cell wall expansion [182,183,184,185], were also downregulated. All of these genes were found in the DEGs of 14-day-old seedlings sealed with PE plastic wrap and PP parafilm (CK14d vs. PW14d, CK14d vs. PF14d), and *AT4G18780 (CESA8)* also appeared in the DEGs of 7-day-old seedlings under the same conditions. *AT3G50220 (IRX15)* also appeared in the DEGs of 7-day-old seedlings sealed with PE plastic wrap (Supplementary Data Set 2). None of these genes were differentially expressed in NF surgical tape-sealed dishes, consistent with the observation that seedlings sealed with NF surgical tape grew similarly to unsealed ones.

**Figure 9:**
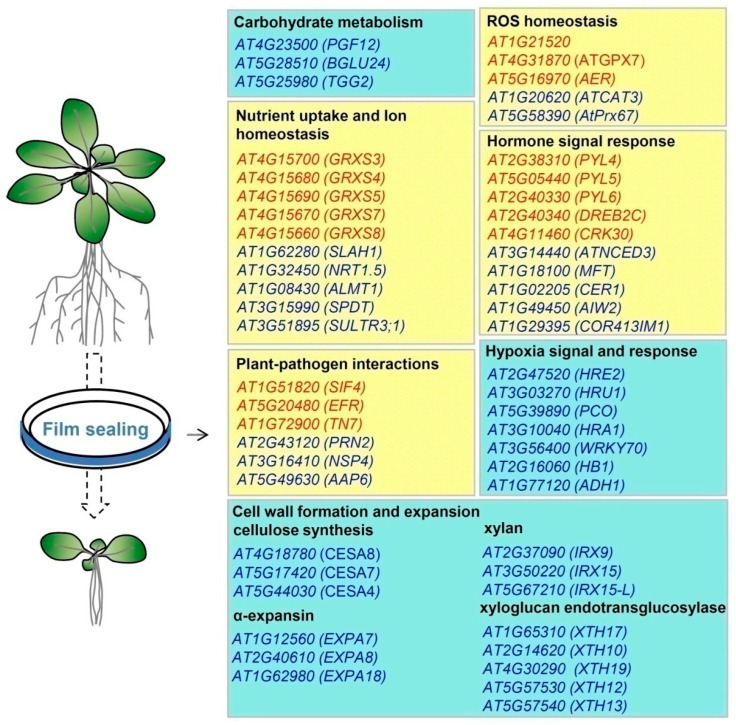
Representative DEGs in film-sealed dishes. The yellow background rectangle box includes both upregulated and downregulated genes, while the blue background rectangle box only includes downregulated genes. Red font represents upregulated genes, and blue font represents downregulated genes.

In summary, sealing conditions, particularly with PE plastic wrap and PP parafilm, create a stress environment with reduced CO_2_ content, enhancing photorespiration. Seedlings grown under these conditions exhibit delayed growth and reduced size compared to those grown in unsealed dishes over the same period. Genes involved in growth, development, stress and hormone responses, pathogen interactions, and ion homeostasis were both up-and down-regulated, contributing to the observed phenotype of compromised growth (Figure 9).

Of course, the reason for the compromised growth of plants caused by film sealing may not be limited to the decrease in CO_2_ concentration, which leads to a series of related gene expression changes. However, film sealing did induce a substantial number of differential expressions of genes compared to unsealed seedlings. Therefore, it is advisable to avoid sealing Petri dishes during *Arabidopsis* seedling growth. Addressing the issue of Petri dish sealing is crucial for ensuring scientific rigor and reliability in experimental outcomes.

## 4. Materials and Methods

### 4.1. Plant Growth Conditions and Sample Preparation

*Arabidopsis thaliana* (L.) Heynh. ecotype Columbia (Col-0) was used. Seeds were surface-sterilized with a 4:1 mixture of 85% ethanol and 30% hydrogen peroxide for 30 s to 1 min and then washed five times with sterilized water. Sixty seeds were evenly distributed on each Petri dish containing agar-based Murashige and Skoog (MS) medium. PP parafilm, PE plastic wrap, and NF surgical tape were used to seal the Petri dish with 0, 1/2, 1, and 2 layers. After cold treatment (4 °C) in the dark for 3–5 days, Petri dishes were transferred to a growth chamber under a 16h light/8h dark cycle at 22 °C for 7 and 14 days. To investigate diameter and fresh weight, 50 seedlings from the same Petri dish were measured for each treatment, except for measuring 25 seedlings in half of the sealed Petri dish. Each treatment was performed on three biological replicates. The diameter refers to the sum of the lengths of the two largest leaves.

PP parafilm (PM-996) was purchased from Amcor (New Albany, Ohio, America). NF surgical tape was from 3M Japan Innovation Limited (Tokyo, Japan). PE plastic wrap was produced by the Top Daily Chemicals China Co., Ltd. (Suqian, Jiangsu Province, China). The temperature tolerance range of this plastic wrap was −60 to 110 °C. The length and width of sealing films required for each circle are 5.1 and 1.3 cm, respectively.

### 4.2. RNA Isolation

Plants were sampled during two growth periods (7 and 14 days) and four sealing conditions (PP Parafilm, PE plastic wrap, and NF surgical tape sealed with one layer and unsealed Petri dishes). Each sample was composed of fifty intact *Arabidopsis* seedlings grown in one Petri dish with three biological replicates. The samples were immediately frozen in liquid N_2_ and ground into powder. Total RNA was extracted using the TRIpure Reagent (Aidlab) according to the manufacturer’s instructions. It mainly included the following steps. After thoroughly mixing the sample powder with TRIzol, we added chloroform and shook vigorously to mix well. Next, the samples were centrifuged at 4 °C with 12,000 rpm for 15 min, and the upper aqueous layer was transferred into a new tube and added with an equal volume of isopropanol to precipitate RNA. After washing with 75% ethanol, the precipitated RNA was dried and dissolved with RNase-free water.

### 4.3. RNA Library Preparation and Sequencing

The operation was carried out according to the BGI Optimal mRNA Library Construction Kit (BGI-Shenzhen, China). Three hundred nanograms of RNA samples were denatured at a suitable temperature to open their secondary structure, and mRNA was enriched by oligo(dT)-attached magnetic beads. After mRNA fragmentation, double-stranded cDNA was synthesized and further repaired by adding base A to the *3′* terminus. Afterwards, the adapter was connected to cDNA, and the product was amplified by PCR. After denaturing the PCR product into a single chain, perform a cyclization reaction to acquire single-stranded cyclized products. Next, single-stranded circular DNA molecules were replicated via rolling circle amplification, and a DNA nanoball (DNB), which contains multiple copies of DNA, was generated. Sufficient quality DNBs were then loaded into patterned nanoarrays using a high-intensity DNA nanochip technique.

The fragmented reads (2×150bp) with insert sizes of 300 bp for paired-end reads were produced using the DNBSEQ platforms (BGI-Tech, Wuhan, China). The raw data were filtered using SOAPnuke to remove reads containing adapters, reads with an unknown base “N” content greater than 0.1%, and low-quality reads (reads with a percentage of bases with a quality value less than 15 exceeding 20% of the total bases in the read). The filtered data, referred to as clean data, were subsequently analyzed. Each sample yielded an average of 6.73 GB of clean data. The clean reads were compared to reference genome sequences (Arabidopsis_thaliana_3702.arabidopsis.TAIR10.v2201) using HISAT and Bowtie2. Fragments Per Kilobase of exon model Per Million mapped Fragments (FPKM) ≥ 1 was used to obtain the expressed genes and generate the PCA. To assess the gene expression level and abundance of the sample, TPM was calculated to normalize the gene length and sequencing depth, and log-function normalization of clean data were performed according to log_10_ (TPM + 1). The fold changes between the experimental group and the control group were calculated. The differentially expressed genes (DEGs) were identified using DESeq2 with criteria set as Q value ≤ 0.05 and log_2_ fold change ≥ 1.

### 4.4. Functional Annotation

Gene ontology (GO) was performed using the Dr. Tom system of BGI Genomics Co., Ltd. (Shenzhen, China) Enrichment analysis was performed using the hyper function in R scripting front-end version 3.5.1 (accessed on 2 July 2018) to calculate *p*-values. FDR correction was applied to the *p*-values to obtain Q-values. According to the criterion of Q-value < 0.05, whether DEGs are significantly enriched in GO items was detected. All expressed genes in both the control group and the experimental group were used to perform Gene Set Enrichment Analysis (GSEA). The “clusterProfiler” R package was used for GSEA. Pathways with a normal *p*-value < 0.05 and a false discovery rate < 0.25 were considered significantly enriched.

### 4.5. Gas Content Measurement

The content of O_2_ and CO_2_ in the Petri dish of 7- and 14-day-old seedlings grown in it was measured by the gas detector tube (GASTEC, Tokyo, Japan). We inserted the detector tube into the sampling pump and extracted 50 mL of gas from the Petri dish to test. After reacting with gas in the detection layer for 2 min, the color of the detector tube will change, and data can be read based on the corresponding scale [186]. The measuring range is 6~24% of the O_2_ detector tube (No. 31B); the color changes from black to white, corresponding to specific O_2_ content values. The reaction principle is O_2_ + 4TiCl_3_ + 6H_2_O → 4TiO_2_ + 12HCl. The measuring range is 100~2000 ppm of the CO_2_ detector tube (No. 2LC); the color change from pale red to orange corresponds to a specific value, similar to the O_2_ detector tube. The reaction principle is CO_2_ + 2KOH → K_2_CO_3_ + H_2_O. Each treatment was repeated at least three times.

### 4.6. qRT-PCR Detection

RNA was reverse transcribed with TRUEscript RT MasterMix (Aidlab) to synthesize single-stranded cDNA. The cDNA was amplified by quantitative real-time PCR (qRT-PCR) with the Taq Pro Universal SYBR qPCR Master Mix (Vazyme) and carried out using the Quant Studio Real Time PCR detection system. *Actin* was used as internal reference. The relative gene expression was calculated by the quantitative method (2^−ΔΔCt^) [187]. Each treatment consisted of three technical replicates and two biological replicates. The gene-specific primers used in qRT-PCR were listed in Table S2.

## Figures and Tables

**Table 1:** Statistics of differentially expressed genes (DEGs).

Catalog	DEGs	Up	Down
CK7d vs. PW7d	1814	643	1171
CK7d vs. PF7d	1083	635	448
CK7d vs. ST7d	2	2	0
CK14d vs. PW14d	2644	592	1818
CK14d vs. PF14d	2410	644	2000
CK14d vs. ST14d	2246	751	1495
CK7d vs. PW7d and CK7d vs. PF7d	609	274	335
CK14d vs. PW14d and CK14d vs. PF14d	1614	347	1267
CK7d vs. PW7d and CK7d vs. PF7d and CK14d vs. PW14d and CK14d vs. PF14d	220	73	134

Note: “CK7d vs. PW7d and CK7d vs. PF7d” represented that DEGs appeared in both “CK7d vs. PW7d” and “CK7d vs. PF7d”. “CK14d vs. PW14d and CK14d vs. PF14d” represented that DEGs appeared in both “CK14d vs. PW14d” and “CK14d vs. PF14d”. “CK7d vs. PW7d and CK7d vs. PF7d and CK14d vs. PW14d and CK14d vs. PF14d” represented the simultaneous occurrence of DEGs in groups “CK7d vs. PW7d”, “CK7d vs. PF7d”, “CK14d vs. PW14d”, and “CK14d vs. PF14d”.

**Table 2:** In total, 51 upregulated genes related to compromised growth under PE plastic wrap and PP parafilm conditions.

Gene ID	Other Name	Description	Functional Classification	Representative Reference	log_2_ (CK7d vs. PW7d)	log_2_ (CK7d vs. PF7d)	log_2_ (CK14d vs. PW14d)	log_2_ (CK14d vs. PF14d)
*AT1G21520*		hypothetical protein	Stress response (oxidative stress)	[6,7]	2.28	2.74	1.23	2.22
*AT4G31870*	*ATGPX7*	Glutathione peroxidase	Stress response (oxidative stress)	[8,9,10]	2.55	1.98	1.91	1.65
*AT5G16970*	*AER*	A 2-alkenal reductase (EC 1.3.1.74)	Stress response (oxidative stress)	[11,12]	1.87	1.79	1.17	1.03
*AT1G10585*		Basic helix-loop-helix (bHLH) DNA-binding superfamily protein	Stress response (oxidative stress and wounding stress)	[13]	2.31	3.82	1.94	3.05
*AT3G62930*	*ROXY17*/*AtGRXS6*	Member of the CC-type glutaredoxin (ROXY) family	Stress response (oxidative stress) + Nutrient uptake (nitrate)	[14,15]	2.22	1.65	1.84	1.24
*AT1G22400*	*UGT85A1*	UDP-Glycosyltransferase superfamily protein	Stress response (heat)	[16,17]	1.04	1.58	1.71	1.72
*AT5G03720*	*HSFA3*	Member of heat stress transcription factor (Hsf) family	Stress response (heat)	[18,19]	2	1.18	2.2	1.72
*AT2G04050*		MATE efflux family protein	Stress response (Salt)	[20]	5.94	4.15	2.12	3.51
*AT2G38310*	*PYL4*	Member of the PYR (pyrabactinresistance)/PYL(PYR1-like)/RCAR (regulatory components of ABA receptor) family proteins	Hormone response (abscisic acid)	[21]	1.25	1.59	1.57	1.16
*AT5G05440*	*PYL5*	Member of the PYR (pyrabactinresistance)/PYL(PYR1-like)/RCAR (regulatory components of ABA receptor) family proteins	Hormone response (abscisic acid)	[21]	1.93	2.07	1.63	1.59
*AT2G40330*	*PYL6*	Member of the PYR (pyrabactinresistance)/PYL(PYR1-like)/RCAR (regulatory components of ABA receptor) family proteins	Hormone response (abscisic acid)	[22]	1.8	2.32	2.82	2.65
*AT4G16563*		Eukaryotic aspartyl protease family protein	Hormone response (abscisic acid)	[23]	1.51	1.4	2.16	2.2
*AT4G11460*	*CRK30*	Cysteine-rich receptor-like protein kinase	Hormone response (abscisic acid)	[24]	1.23	2.68	6.7	6.45
*AT2G40340*	*DREB2C*	Member of the DREB subfamily A-2 of ERF/AP2 transcription factor family	Hormone response (abscisic acid)	[25]	2.57	1.82	2.3	2.65
*AT3G14620*	*CYP72A8*	Putative cytochrome P450	Hormone response (phytoprostanes)	[26]	1.68	1.94	2.15	1.77
*AT5G16980*		Zinc-binding dehydrogenase family protein	Hormone response (phytoprostanes)	[26]	3.13	2.59	2.33	2.03
*AT5G60250*		Zinc finger (C3HC4-type RING finger) family protein	Hormone response (cytokinin)	[27]	5.25	1.6	3.91	1.95
*AT1G35140*	*EXL1*	Exordium like 1; Hypoxia response unknown protein 46	Stress response (carbon starvation) + Hormone response (brassinolide)	[28,29]	2.78	2.43	2.4	2.43
*AT5G13210*		Uncharacterized conserved protein UCP015417	Stress response (sucrose starvation)	[30]	2.24	1.01	1.2	1
*AT3G01970*	*WRKY45*	Member of WRKY Transcription Factor	Stress response (phosphate starvation)	[31]	1.98	2.02	1.28	1.73
*AT4G34950*	*MFS1*	Major facilitator superfamily protein	Stress response (phosphate starvation)	[32]	1.28	1.68	1.29	1.52
*AT2G04040*	*ATDTX1*	A detoxifying efflux carrier for plant-derived antibiotics and other toxic compounds	Stress response (heavy metal, Cadmium)	[33]	3.04	4.23	3.32	4.08
*AT1G51820*	*SIF4*	Leucine-rich repeat protein kinase family protein	Plant-pathogen interactions	[34]	1.41	2.51	1.48	2.04
*AT1G63750*		MiR825-5p target proposed as a phasiRNA producing locus	Plant-pathogen interactions	[35]	1.35	1.05	1.44	1.19
*AT1G72900*	*TN7*	Toll-Interleukin-Resistance (TIR) domain-containing protein	Plant-pathogen interactions	[36]	1.7	2.17	2.02	2.3
*AT2G32550*	*NOT9C*	Rcd1-like protein	Plant-pathogen interactions	[37]	2.9	1.3	2.57	1.94
*AT4G25110*	*AtMC2*	A type I metacaspase	Plant-pathogen interactions	[38]	1.77	2.13	2.02	2.23
*AT5G11410*	*SZE2*	Similar to receptor like kinase but does not appear to have kinase activity (pseudokinase)	Plant-pathogen interactions	[39]	3.19	2.52	2.12	2.32
*AT5G20480*	*EFR*	A predicted leucine-rich repeat receptor kinase (LRR-RLK)	Plant-pathogen interactions	[40]	1.17	1.82	1.25	1.29
*AT4G15660*	*GRXS8;ROXY15*	A member of the CC-type glutaredoxin (ROXY) family	Nutrient uptake (soil nitrate)	[41]	4.74	5.41	1.71	2
*AT4G15670*	*GRXS7;ROXY14*	A member of the CC-type glutaredoxin (ROXY) family	Nutrient uptake (soil nitrate)	[42]	3.23	3.88	3.91	4.76
*AT4G15680*	*GRXS4;ROXY13*	A member of the CC-type glutaredoxin (ROXY) family	Nutrient uptake (soil nitrate)	[42]	3.36	3.5	3.05	3.44
*AT4G15690*	*GRXS5;ROXY12*	A member of the CC-type glutaredoxin (ROXY) family	Nutrient uptake (soil nitrate)	[42]	3.34	3.46	1.81	2.3
*AT4G15700*	*GRXS3;ROXY11*	A member of the CC-type glutaredoxin (ROXY) family	Nutrient uptake (soil nitrate)	[42]	3.58	3.97	1.71	2.37
*AT1G09240*	*NAS3*	A nicotianamine synthase	Nutrient uptake (iron)	[43,44]	2.05	1.07	1.66	1.14
*AT1G20350*	*TIM17-1*	Mitochondrial inner membrane translocase	Growth and development (seed germination)	[45]	4.5	2.57	2.25	2.03
*AT2G30140*	*UGT87A2*	A putative glycosyltransferase	Growth and development (flowering time)	[46]	1.07	1.52	1.07	1.6
*AT2G36750*	*UGT73C1*	UDP-glucosyl transferase 73CC1	Growth and development (pistils and siliques elongation)	[47]	2.91	3.48	1.65	2.92
*AT3G04000*	*CHLADR*	An aldehyde reductase	Growth and development (seedlings growth)	[48]	1.52	1.81	1.84	1.62
*AT5G13370*	*GH3.15*	IBA—specific acyl acid amido synthetase, which conjugates glutamine to IBA	Growth and development (primary root and lateral root growth)	[49,50]	1.53	1.52	1.19	1.3
*AT5G16960*		Zinc-binding dehydrogenase family protein	Growth and development (pollen tube growth)	[51]	3.05	4.85	1.6	3.33
*AT1G20120*	*AtGELP3*	GDSL-motif esterase/acyltransferase/lipase	Unknown	[52]	4.35	2.37	1.48	1.16
*AT2G29480*	*ATGSTU2*	Glutathione transferase belonging to the tau class of GSTs	unknown	[53]	1.85	3.31	1.07	1.56
*AT1G35350*	*PHO1;H8*	EXS (ERD1/XPR1/SYG1) family protein	Unknown	[54]	1.04	1.53	1.2	1.26
*AT2G23100*		Cysteine/Histidine-rich C1 domain family protein	Unknown	[55]	2.11	1.37	2.04	1.84
*AT2G29370*		NAD(P)-binding Rossmann-fold superfamily protein	Unknown	none	2.11	1.8	3.6	3.39
*AT2G30615*		F-box/LRR protein	Unknown	none	3.35	2.96	1.73	2.09
*AT3G21530*		DNAse I-like superfamily protein	Unknown	none	1.29	1.67	1.67	1.8
*AT3G26320*	*CYP71B36*	Putative cytochrome P450	Unknown	none	1.11	1.22	1.27	1.56
*AT5G55570*		Transmembrane protein	Unknown	[56]	2.16	1.31	1.45	1.04
*AT5G54710*		Ankyrin repeat family protein	Unknown	none	1.38	2.25	2.23	2.03

Note: Genes were classified according to the column “functional classification”, and genes with the same functional classification were ranked from small to large according to the order on the chromosome.

**Table 3:** In total, 65 downregulated genes related to compromised growth under PE plastic wrap and PP parafilm conditions.

Gene ID	Other Name	Description	Functional Classification	Representative Reference	log_2_ (CK7d vs. PW7d)	log_2_ (CK7d vs. PF7d)	log_2_ (CK14d vs. PW14d)	log_2_ (CK14d vs. PF14d)
*AT2G22240*	*ATMIPS2*	Myo-inositol-1-phosphate synthase isoform 2	Growth and development (vegetative growth)	[57]	−1.09	−1.29	−1.85	−1.69
*AT3G49960*	*PRX35*	Class III peroxidase genes	Growth and development (vegetative growth)	[58]	−1.51	−1.33	−3.27	−4.44
*AT4G18780*	*CESA8/IRX1*	A member of the cellulose synthase family	Growth and development (vegetative growth)	[59,60]	−1.51	−1.08	−1.86	−2.32
*AT2G33385*	*ARPC2B*	Actin-related protein C2B	Growth and development (cell morphogenesis)	[61]	−2.79	−1.67	−1.32	−1.98
*AT3G21330*		Basic helix-loop-helix (bHLH) DNA-binding superfamily protein	Growth and development (seedling development)	[62,63]	−2.65	−1.42	−1.91	−1.23
*AT5G50800*	*SWEET13*	Member of the SWEET sucrose efflux transporter family proteins	Growth and development (anthers, seeds and seedlings development)	[64,65]	−4.66	−2.87	−4	−3.56
*AT5G54190*	*PORA*	Light-dependent NADPH:protochlorophyllide oxidoreductase A	Growth and development (photomorphogenesis) + Stress response (oxidative stress)	[66,67]	−2.33	−2.15	−2.6	−1.06
*AT5G15160*	*BNQ2*	A atypical non-DNA binding basic helix-loop-helix (bHLH) proteins	Growth and development (hypocotyl elongation, petal differentiation and flowering time)	[68]	−1.71	−1.24	−2.31	−1.96
*AT4G32280*	*IAA29*	Indole-3-acetic acid inducible 29 protein	Growth and development (hypocotyl growth)	[69]	−1.79	−1.64	−4.31	−3.14
*AT1G04180*	*YUC9*	Flavin monooxygenase	Growth and development (hypocotyl and root growth)	[70,71]	−3.02	−2.58	−1.72	−1.94
*AT1G12110*	*NRT1.1/CHL1*	A dual-affinity nitrate transporter.	Growth and development (lateral root development)	[72,73]	−1.15	−1.16	−2.17	−2
*AT2G39370*	*MAKR4*	A member of the MAKR (MEMBRANE-ASSOCIATED KINASE REGULATOR) gene family	Growth and development (lateral roots formation)	[74]	−1.32	−2.14	−1.7	−2.35
*AT3G27150*	*HOLT*	Target gene of MIR2111-5p	Growth and development (lateral root initiation)	[75]	−1.17	−1.22	−1.26	−1.26
*AT4G30290*	*XTH19*	A xyloglucan endotransglucosylase/hydrolase	Growth and development (lateral root development)	[76,77]	−1.05	−1.1	−1.58	−1.92
*AT3G48340*	*CEP2*	KDEL-tailed cysteine endopeptidase	Growth and development (lateral root growth)	[78,79]	−1.8	−1.06	−2.81	−2.84
*AT1G66470*	*RHD6*	Basic helix loop helix transcription factor	Growth and development (root hair initiation)	[80,81]	−1.6	−1.34	−1.52	−1.71
*AT4G34580*	*COW1/AtSFH1*	A phosphatidylinositol transfer protein	Growth and development (root hair growth)	[82,83]	−2.19	−1.1	−2.15	−1.79
*AT1G18100*	*MFT*	A member of the FT and TFL1 family of phosphatidylethanolamine-binding proteins	Growth and development (seed germination) + Hormone response (abscisic acid)	[84]	−1.36	−1.71	−2.74	−3.1
*AT2G18980*	*PRX16*	Class III peroxidase cell wall-targeted protein	Growth and development (seed germination) + Stress response (salt)	[85,86,87]	−1.99	−1.01	−1.6	−2.38
*AT1G18710*	*ATMYB47*	Member of the R2R3 factor gene family	Growth and development (seed longevity) + Stress response (drought)	[88,89]	−3.25	−1.45	−2.97	−1.23
*AT1G62710*	*AtAEP2; β-VPE*	A vacuolar processing enzyme	Growth and development (seed proteins maturation)	[90,91]	−2.11	−1.46	−2.39	−1.62
*AT1G56710*	*PGL1*	Pectin lyase-like superfamily protein	Growth and development (anther morphogenesis)	[92]	−2.58	−2	−2.11	−2.33
*AT1G13280*	*AOC4*	An allene oxide cyclase	Growth and development (male fertility)	[93]	−1.07	−1.04	−1.14	−1.25
*AT1G02205*	*CER1*	An aldehyde decarbonylase	Growth and development (male fertility) + Hormone response (abscisic acid)	[94,95]	−1.04	−1.14	−2.06	−2.08
*AT4G36880*	*AtCP1*	Cysteine proteinase1	Hormone response (gibberellin)	[96]	−2.76	−1.56	−2.13	−2.2
*AT3G12500*	*PR3*	A basic chitinase	Hormone response (ethylene/jasmonic acid)	[97]	−2.71	−2.12	−2.85	−2.77
*AT5G39190*	*GER2*	Germin-like protein	Hormone response (ethylene)	[98]	−3.6	−1.65	−2.63	−2.26
*AT5G52320*	*CYP96A4*	Cytochrome P450 enzyme	Hormone response (jasmonic acid)	[99]	−3.25	−1.5	−2.34	−1.69
*AT1G78490*	*CYP708A3*	Member of CYP708A family	Hormone response (brassinolide)	[100]	−2.76	−1.48	−1.6	−1.19
*AT4G39510*	*CYP96A12*	Member of CYP96A	Hormone response (brassinolide)	[100]	−1.39	−1.4	−1.55	−1.3
*AT3G22540*		Hypothetical protein	Hormone response (cytokinin)	[27]	−1.09	−1.33	−1.04	−1.73
*AT5G45080*	*PP2-A6*	Phloem protein 2-A6	Hormone response (cytokinin)	[27]	−2.08	−1.56	−1.6	−1.52
*AT1G49450*	*AIW2*	Transducin/WD40 repeat-like superfamily protein	Hormone response (abscisic acid)	[101]	−1.38	−1.38	−2.12	−1.87
*AT1G29395*	*COR413IM1;COR414-TM1*	Integral membrane protein in the inner envelope of chloroplasts	Hormone response (abscisic acid) + Stress response (drought and cold)	[102,103,104]	−4.33	−2.55	−4.79	−3.41
*AT3G14440*	*ATNCED3*	Encodes 9-cis-epoxycarotenoid dioxygenase	Hormone response (abscisic acid) + Stress response (drought and salt)	[105]	−1.51	−1.29	−3.59	−1.69
*AT1G20620*	*ATCAT3*	Catalase	Stress response (oxidative stress)	[106]	−2.12	−1.34	−2.2	−1.16
*AT5G58390*	*AtP44/AtPrx67*	Peroxidase superfamily protein	Stress response (oxidative stress)	[107,108,109]	−3.67	−1.65	−1.77	−1.23
*AT1G20450*	*ERD10*	Encodes a gene induced by low temperature and dehydration	Stress response (salt, drought, and cold)	[110]	−1.11	−1.25	−1.49	−1.17
*AT2G37900*		Major facilitator superfamily protein	Stress response (heavy metal, Cadmium)	[111]	−3.46	−1.79	−2.08	−1.67
*AT4G23500*	*PGF12*	Pectin lyase-like superfamily protein	Stress response (salt) + Carbohydrate metabolic	[112,113]	−1.66	−1.44	−1.7	−1.52
*AT1G62280*	*SLAH1*	A protein with ten predicted transmembrane helices	Ion translocation + Nutrient uptake (potassium, chloride, malate, fumarate, and succinate)	[114,115]	−2	−1.45	−2.32	−3.36
*AT1G32450*	*NRT1.5;NPF7.3*	Transmembrane nitrate transporter	Ion translocation (potassium and nitrate)	[116,117]	−1.15	−1.19	−1.36	−1.25
*AT1G08430*	*ALMT1*	Al-activated malate efflux transporter	Nutrient uptake (aluminum-activated malate)	[118]	−1.76	−2.35	−1.94	−2.29
*AT5G28510*	*BGLU24*	Beta glucosidase 24	Ion translocation (potassium) + Carbohydrate metabolic	[119,120]	−5.6	−5.61	−4.92	−3.91
*AT3G15990*	*SPDT*	Vascular cambium-localized sulfate transporter	Ion translocation (phosphorus)	[121]	−2.07	−1.42	−1.6	−1.2
*AT3G51895*	*SULTR3;1*	Encodes a chloroplast-localized sulfate transporter	Ion translocation (sulfate)	[122]	−3.19	−2.14	−2.95	−1.73
*AT1G09350*	*ATGOLS3*	Predicted to encode a galactinol synthase	Plant-pathogen interactions + Stress response (cold)	[123,124]	−3.94	−2.76	−3.04	−3.02
*AT2G43120*	*PIRIN2*	A member of the functionally diverse cupin protein superfamily	Plant-pathogen interactions	[125]	−2.24	−1.02	−1.96	−1.9
*AT3G16410*	*NSP4*	Encodes a nitrile-specifier protein NSP4	Plant-pathogen interactions	[126]	−2.4	−1.24	−1.7	−2.08
*AT5G25980*	*TGG2*	Myrosinase (thioglucosideglucohydrolase) gene	Plant-pathogen interactions + Carbohydrate metabolic	[127,128]	−3.19	−1.19	−3.29	−2.39
*AT5G49630*	*AAP6*	A high affinity amino acid transporter	Plant-pathogen interactions	[129,130]	−3.12	−1.89	−2.64	−2.73
*AT2G17060*		Disease resistance protein (TIR-NBS-LRR class) family	Pseudogenes	[131]	−1.35	−1.57	−1.47	−1.6
*AT1G51870*		Protein kinase family protein	unknown	none	−2.67	−1.71	−1.71	−2.24
*AT1G66830*		Leucine-rich repeat protein kinase family protein	unknown	[132]	−2.66	−1.89	−1.27	−1.66
*AT1G80240*	*DGR1*	DUF642 gene	unknown	[133]	−1.29	−1.21	−2.03	−3.04
*AT2G18480*	*AtPLT3*	Polyol transporter	unknown	[134]	−2.05	−2.54	−1.62	−1.52
*AT2G28780*		P-hydroxybenzoic acid efflux pump subunit	unknown	[135]	−2.27	−2.42	−1.65	−2.11
*AT3G08660*		Phototropicresponsive NPH3 family protein	unknown	none	−1.37	−1.34	−1.91	−1.25
*AT3G53980*		Bifunctional inhibitor/lipid-transfer protein/seed storage 2S albumin superfamily protein	unknown	[136]	−1.74	−1.37	−3.59	−3.35
*AT5G38100*		SABATH family methyltransferase	unknown	[137]	−1.99	−1.29	−1.04	−1.26
*AT5G45850*		Hypothetical protein (DUF688)	unknown	[138]	−2.62	−2.16	−1.92	−1.17
*AT5G47000*	*AtP43/AtPrx65*	Peroxidase superfamily protein	unknown	[107,109]	−1.83	−2.36	−2.43	−2.42
*AT5G49120*		DUF581 family protein, putative (DUF581)	unknown	[139]	−2.85	−1.99	−1.98	−1.02
*AT5G51750*	*SBT1.3*	Subtilase 1.3	unknown	[140]	−1.07	−1.01	−1.01	−1.03
*AT5G60650*		Proline-rich receptor-like kinase	unknown	none	−1.76	−1.38	−1.42	−1.22

Note: The ranking order of the genes was the same as in Table 2.

## Data Availability

All transcriptome raw sequence data are under NCBI BioProject Accession PRJNA1197163.

## Supplementary Materials

The following supporting information can be downloaded at: https://www.mdpi.com/article/10.3390/ijms26125484/s1.

## Abbreviations

The following abbreviations are used in this manuscript:

DEGs	differentially expressed genes
RNA-seq	transcriptome sequencing
PE	polyethylene
PP	polyolefin and paraffin wax
NF	non-woven fabric and plastic
PW	PE plastic wrap
PF	PP parafilm
ST	NF surgical tape
PCA	principal component analysis
BP	biological processes
MS	Murashige and Skoog
qRT-PCR	quantitative real-time PCR
