# Supplementary material for: Beware of Sealing Film of Petri Dishes!—Alters the Expression of a Large Number of Genes
Source: Int J Mol Sci. 2025 Jun 7;26(12):5484. doi: 10.3390/ijms26125484 (PMC12193506; doi:10.3390/ijms26125484)
Supplement: Supplementary file 1 [file ijms-26-05484-s001.zip › Figure S1.pdf]

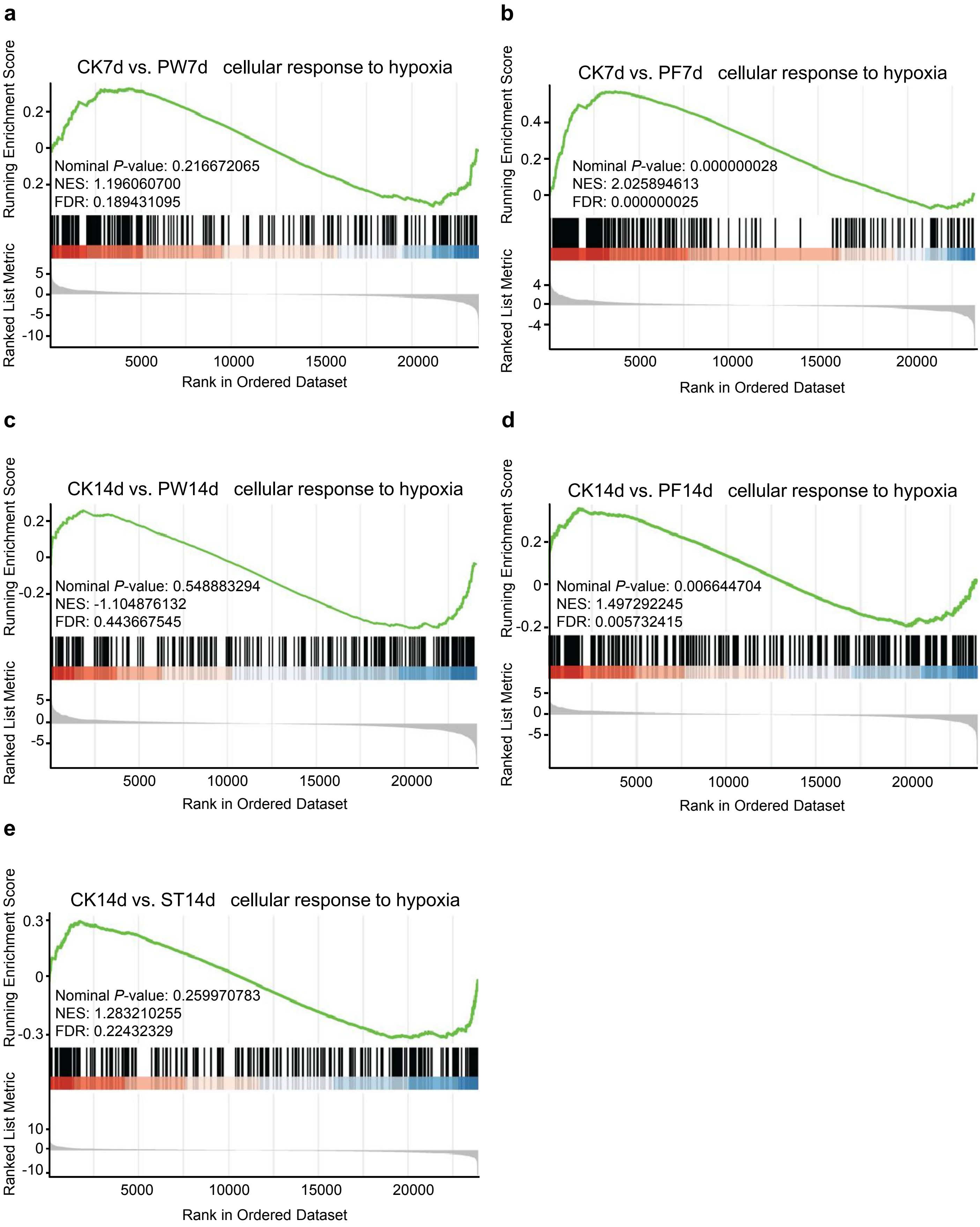

Figure S1. Enrichment of cellular response to hypoxia by gene set enrichment analysis (GSEA).
(a) CK7d vs. PW7d , **(b)** CK7d vs. PF7d , **(c)** CK14d vs. PW14d , **(d)** CK14d vs. PF14d , and **(e)** CK14d vs. ST14d .
